# Multiorgan Failure and Sepsis in an ICU Patient with Prolidase Enzyme Deficiency—The Specificity of Treatment and Care: A Case Report

**DOI:** 10.3390/medicina60061006

**Published:** 2024-06-20

**Authors:** Katarzyna Wojnar-Gruszka, Ilona Nowak-Kózka, Jakub Cichoń, Aleksandra Ogryzek, Lucyna Płaszewska-Żywko

**Affiliations:** 1 Department of Clinical Nursing, Faculty of Health Sciences, Jagiellonian University—Medical College, 31-501 Kraków, Poland; k.wojnar-gruszka@uj.edu.pl (K.W.-G.); lucyna.plaszewska-zywko@uj.edu.pl (L.P.-Ż.); 2 Center for Innovative Medical Education, Jagiellonian University—Medical College, 30-688 Kraków, Poland; ilona.nowak@uj.edu.pl; 3 Clinical Department of Neurology, University Hospital in Kraków, 30-688 Kraków, Poland

**Keywords:** prolidase deficiency, pain, sepsis, multiorgan failure, nursing care, intensive care unit

## Abstract

*Background and Objectives*: Prolidase deficiency (PD) is a rare, life-threatening, genetically determined disease with an incidence of 1–2 cases per 1 million births. The disease inhibits collagen synthesis, which leads to organ and systems failure, including hepato- and splenomegaly, immune disorders, chronic ulcerative wounds, respiratory infections, and pulmonary fibrosis. The complexity of the problems associated with this disease necessitates a comprehensive approach and the involvement of an interdisciplinary team. The objective was to present the treatment and care plan, as well as complications of PD, in a young woman following admission to an intensive care unit (ICU). *Materials and Methods*: A retrospective observational single-case study. *Results*: A 26-year-old woman with PD was hospitalized in the ICU for acute respiratory failure. The presence of difficult-to-heal extensive leg ulcers and the patient’s immunocompromised condition resulted in the development of sepsis with multiple organ failure (respiratory and circulatory, liver and kidney failure). Complex specialized treatment consisting of wound preparation, limb amputation, the minimization of neuropathic pain, mechanical ventilation, renal replacement therapy, circulatory stabilization, and the prevention of complications of the disease and of therapy were applied. On the 83rd day of hospitalization, the patient expired. *Conclusions*: Despite the use of complex treatment and care, due to the advanced nature of the disease and the lack of therapies with proven efficacy, treatment was unsuccessful. There is a need for evidence-based research to develop effective treatment guidelines for PD.

## 1. Introduction

Prolidase deficiency (PD) is a genetically determined metabolic disorder, inherited in an autosomal recessive manner, caused by aberrations in the PEPD gene and leading to the abolition of prolidase enzyme activity [1,2]. Currently, the exact number of PD patients worldwide is unknown. The incidence is estimated to be 1–2 cases per 1 million births and is higher in some populations, including Druze minorities and Arab Muslims in Israel [3,4]. Prolidase deficiency results in the increased excretion of proline and hydroxyproline, which leads to abnormal collagen synthesis [5]. Prolidase thus plays an important role in inflammation, angiogenesis, wound healing, and carcinogenesis. Most patients present their first symptoms in early childhood [3,4]. Patients with PD often present with painful and difficult-to-treat ulcers, most often on the lower extremities [4], eczema or scaly, maculopapular, erythematous, or other inflammatory lesions (AD—atopic dermatitis). Telangiectasia is a common symptom [3,6,7]. Most patients have facial dysmorphic features, including widely spaced eyes (hypertelorism), deep-set eyes, low-set ears, saddle nose, synophrys, a low hairline, narrow upper lip, or prognathism [3,8,9]. In addition, low stature, microcephaly, osteopenia, and valgus knees may be present [10].

Patients with PD may also present with gastrointestinal symptoms, such as splenomegaly (about 75% of diagnosed patients), hepatomegaly (in about 50%) and, less commonly, gastrointestinal ulcers and inflammatory lesions [3]. In addition, systemic lupus erythematosus, thrombocytopenia, hypergammaglobulinemia, anemia, thrombocytopenia and elevated liver enzymes may be present [3,6,7,9]. Respiratory disorders in PD include recurrent respiratory infections, bronchiectasis, cystic lesions, bronchial hyperresponsiveness, and pulmonary fibrosis [3,11], which, despite medical advances, especially in gene therapy, is particularly dangerous and limited to symptomatic treatment [12].

To date, no standard diagnostic criteria for PD have been published. Diagnosis is based on the confirmation of reduced prolidase enzyme activity or a pathogenic variant of the PEPD gene, the finding of imidodipeptiduria, and symptoms characteristic of the condition [4].

Survival data for patients with PD are inconclusive. A review by Rossignol et al. [13] estimated a survival rate of 90% by age 20 and 82% by age 40. The youngest reported death was at the age of three months [14].

Due to the incurable nature of the disease, treatment is focused on symptom relief and the prevention of complications. The patient should be under the care of an interprofessional healthcare team throughout the course of the disease [7]. In special clinical situations resulting from increasing multiorgan failure, treatment requires numerous specialized therapies and supportive treatment.

The complexity of the problems associated with this disease necessitates a comprehensive approach that encompasses a multitude of facets of treatment and care, with the patient’s well-being considered holistically. This requires the involvement of an interdisciplinary team, including physicians, nurses, physiotherapists, clinical pharmacists, and nutritionists, among others. The multidisciplinary approach encompasses a range of patient-related factors, including functional status, comorbidity, nutritional status, polypharmacy, cognitive functions, and psychological state, as well as social support. The team’s activities are based on the recognition of patient needs and the tailoring of interventions and supportive care plans to the individual [15].

Since PD is a rare disease and case reports of patients with this condition are scarce in the literature, we decided to present a case study on the progression of PD requiring hospitalization in an intensive care unit (ICU). The primary goal of our study was to present the treatment and care plan, as well as complications of PD, in a young woman following admission to the ICU.

## 2. Materials and Methods

A retrospective observational single-case study was conducted involving a 26-year-old woman with PD during her two hospitalizations in the intensive care unit (ICU) for respiratory failure and subsequent progressive multiorgan failure. A proprietary observation sheet and data from medical records (including medical history and test results) were analyzed. The RASS (The Richmond Agitation–Sedation Scale) depth of sedation scale, CCPOT (Critical-Care Pain Observation Tool) pain scale, Norton pressure ulcer risk scale, Torrance pressure ulcer severity scale, APACHE II (Acute Physiology and Chronic Health Evaluation II), and SAPS II (Simplified Acute Physiology Score II) scales were also used. The patient was hospitalized in one of the hospitals in southern Poland, between April and June 2019. The study was approved by the Jagiellonian University Bioethics Committee (No. 1072.6120.335.2018). Informed consent for data use and publication was obtained from the patient’s family.

## 3. Results

A 26-year-old patient with PD was admitted to the ICU on an emergency basis from the internal medicine department of a county hospital for increasing respiratory failure.

The patient had a history of PD prior to this ICU admission. Symptoms of ulcers in the lower extremities were first observed when the patient was 14 years old. The patient had been hospitalized several times in the departments of internal medicine, dermatology, and rheumatology. The patient was previously partially independent in activities of daily living. She used crutches due to mobility difficulties. In addition, pain from chronic ulcers with subsequent foul odor caused social problems—avoidance of contact with others, feelings of shame, which exacerbated the symptoms of current depression.

The final diagnosis of PD was made two years prior to admission to the ICU, based on clinical symptoms and laboratory tests. Subsequently, this diagnosis was confirmed by molecular genetic testing, which revealed a mutation in the PEPD gene.

The patient was hospitalized in the ICU for a total of 83 days. Due to the need for Hyperbaric Oxygen Therapy (HBOT) on the 27th day of hospitalization in the original ICU, the patient was transferred to the ICU at another facility with a hyperbaric chamber. After 38 days, the patient was transferred back to the original ICU. In order to present the case report clearly, the authors decided to describe the hospitalizations as ICU 1, ICU 2, and ICU 3.

On admission to ICU 1, the patient was unconscious, intubated by the Helicopter Emergency Medical Service (HEMS), and mechanically ventilated with a FiO_2_ of 0.8, achieving a saturation of 100%. The patient was given a primary diagnosis of Acute Respiratory Distress Syndrome (ARDS), confirmed by imaging and laboratory tests. The patient demonstrated cardiovascular efficiency with the following values: BP 146/90 mmHg, MAP 108 mmHg, HR 112 bpm, capillary return < 3 s, temperature 36.6 degrees C; BMI = 29.41 kg/m^2^. The patient underwent sedation (on the RASS scale from −3 to −5) and analgesia (on the CCPOT scale from 0 to 4), and nutritional treatment was administered. Due to oliguria, the patient required diuretics to achieve fluid–electrolyte balance. Broad-spectrum empirical antibiotic therapy was initiated, followed by targeted antibiotic therapy (Table 1 and Table S1, Figure S1). During the patient’s hospitalization in ICU 1, the skin symptoms clinically manifested as chronic ulcers of the lower extremities, covered by tissue necrosis with a bacterial film, involving joints and bones, with profuse purulent leakage, especially on the left lower limb (Torrance scale grade V; Norton scale 5 points—high risk of developing pressure sores). Dry necrosis was also found in toe III of the right foot. The patient’s skin was dry, parchment-like, and pale with numerous petechiae. Microcytic anemia, hepatosplenomegaly, and hypothyroidism were also diagnosed.

On the 27th day of hospitalization, the patient was transferred to the ICU at another facility with a hyperbaric chamber. During the patient’s 35-day hospitalization, pharmacological treatment and supportive therapies were continued with ICU 1 (MV, analgesia and sedation, circulatory support, antibiotic therapy, antithrombotic prophylaxis, enteral and parenteral nutrition, and CRRT). In addition, two series of HBOT (14 sessions) were performed. During the treatments of HBOT, the patient remained under close ENT control—paracentesis procedures were performed. Debridement on the lower extremities was carried out repeatedly under operating theater conditions. Specialized dressings were applied, including vacuum and biological dressings (larval therapy). Blood products and albumin were administered. Despite systemic and local treatment, high levels of inflammatory parameters persisted throughout the stay in ICU 2. Targeted antibiotic therapy was stopped on day 30 of ICU 2 (of a total 57). An attempt to withhold sedation proved unsuccessful due to significant pain (CCPOT 3–4) and generalized myopathy. Due to the lack of wound healing, the tracheostomy was abandoned despite the prolonged duration of the endotracheal tube. After numerous surgical consultations, it was concluded that options for further limb-sparing management had been exhausted, and that further escalation of surgical treatment was unwarranted. After 38 days in ICU 2, the patient was readmitted to ICU 1 (hereafter referred to as ICU 3) for further treatment. After admission to ICU 3, multidirectional drug treatment and supportive therapies were continued from ICU 2 (MV, tracheostomy was inserted on day 2, analgesia, sedation, and circulatory support). Blood was collected for microbiological studies. Diuretics were initially ordered, but then CVVHD was implemented due to anuria and progressive renal failure. Frequent surgical consultations with dressing changes were performed, including a VAC dressing on the stump of the left limb. On the 8th day of stay, the patient experienced a sudden deterioration in the form of fulminant septic shock with increasing multiorgan failure (Table 1 and Table S1, Figure S2). In the following days, the patient’s clinical condition deteriorated rapidly. Adrenaline infusion was added, and the filtration technique was changed to CVVHDF. The diagnosis of cholecystitis was made, and a cholecystectomy was performed. On the second day after the procedure, the patient’s condition stabilized—doses of pressure amines were reduced, and adrenaline infusion was discontinued. In the following days, despite continuous treatment, the patient’s condition deteriorated. Gradually, multiorgan failure progressed. On day 18, the patient expired.

## 4. Discussion

Prolidase deficiency is a disease fraught with numerous systemic disorders and complications, often ending in premature death. Late diagnosis or inadequate treatment leads to the occurrence of hard-to-heal ulcers, chronic neuropathic pain, systemic disorders, increasing physical disability, and psychological problems [3,4,7,9,13]. Clinical symptoms of PD are highly variable, of varying severity, present in different configurations, and appear in patients of different ages. This is confirmed by Rossignol et al. [13], who provided one of the largest case series of 19 individuals with PD and a review of the literature including 161 patients with PD. For this reason, each case should be considered individually.

In the 26-year-old patient described in this case study, the PD course and its complications were severe. The immediate cause of the patient’s hospitalization in the ICU was acute respiratory failure requiring mechanical ventilation. Respiratory diseases in PD patients are not very well described in the literature. Asthma-like chronic reactive airway disease has been described in approximately 7% of PD patients [7]. Other forms of chronic pulmonary disease are occasionally seen, such as progressive lung disease [16,17], pulmonary hypertension [18], and pulmonary fibrosis [19,20]. In this patient, acute pulmonary infection probably occurred, which, in combination with other lung lesions in the course of PD, led to acute respiratory failure.

The patient had fairly typical ulcers of the lower extremities, which are seen in about 62% of PD patients [3,13]. It is likely that they were untreated prior to hospitalization, or their treatment was ineffective, and that during hospitalization the skin condition deteriorated rapidly, responding poorly to the treatment methods used. To date, no clinical practice guidelines for PD have been published [3,13]. A few studies or case reports suggest a whole range of various treatment attempts, such as topical tacrolimus, topical proline therapy, replacing the deficient enzyme, ointments containing glycine, enzyme replacement therapy, erythrocyte transfusion, immunosuppressive drugs, as well as growth hormone (GH) [3,9,21,22,23].

Unfortunately, the efficacy of the proposed therapies is low, short-lived, insufficiently studied, and some are unavailable [9]. In the absence of guidelines for the treatment of ulcers and other skin lesions in patients with PD, the patient was treated with highly specialized wound therapies. Some of these have been reported in the few case reports of PD patients [3,13,24]. The patient received antibiotic therapy, surgical wound debridement, specialized dressings, negative pressure therapy (VAC), plasmapheresis, anticoagulant therapy, steroid therapy, and sedative and analgesic treatment including an epidural catheter. Hyperbaric chamber treatment was also undertaken because of the promising results of the therapy for hard-to-heal wounds [25,26], including those occurring in PD [27]. The patient required continuous monitoring, repositioning, fluid therapy, nutrition, pain management, renal replacement therapy, the collection of material for numerous tests, and physiotherapy, all of which were extremely time consuming. Due to the complexity of the problems, numerous healthcare professionals participated in the care of the patient, including physicians, nurses, physiotherapists, dietitians, and clinical pharmacists.

The available publications usually describe much less severe cases [21,24,27] or present a combined analysis of symptoms and PD treatments [3,13]. Our case report shows the complexity of therapy and care, which, however, did not have the desired effect.

In our patient the skin lesions worsened, and gangrene of the limb occurred, requiring amputation. The non-healing ulcers that were the gateway to infection, together with the patient’s immunocompromised status and severe anemia, caused the development of septic shock, which, together with the likely pre-existing organ complications of PD, led to multiple organ failure and the death of the patient.

Mortality figures show that, overall, 90% (95% CI 83–94%) of patients are alive by age 20, 88% (95% CI 81–93%) by age 30, and 82% (95% CI 70–90%) by age 40 [13]. It means that the patient presented was among the minority of people with PD in her age group (26 years) whose disease ended in death. It indicates a severe course of the disease and its complications.

The complex therapy and care of the patient required great effort and the cooperation of many professionals (e.g., physicians, nurses, and physiotherapists). Treatment and care management was aimed not only at trying to control sequentially appearing and growing disorders, but also at providing the patient with the highest possible comfort, minimizing pain, preventing complications related to medical and nursing procedures, and preventing further complications of the disease. The individualization of treatment and care, taking into account the patient’s changing clinical condition, made it possible to partially alleviate symptoms, but despite these efforts, the patient’s death occurred. The patient’s case described here illustrates the challenges and difficulties associated with caring for patients with a rare disease in the ICU [28]. Early diagnosis of PD and other rare diseases is not always possible, and the time to diagnosis for many patients is long. Consequently, these diseases are diagnosed at an advanced stage, and treated after the occurrence of complications, as was the case with the patient described. Although knowledge of rare diseases, including PD, continues to increase, it is a major challenge for the interdisciplinary team, mainly because of PD’s chronic and often severe course, difficult diagnosis, lack of effective treatment options, costly symptomatic therapy, and time-consuming, and complex nursing care, requiring the involvement of multiple team members due to complications and hospitalizations.

Undertaking research on therapies for PD and other rare diseases, disseminating knowledge about them, improving detection, and developing guidelines and strategies for the management of rare diseases are important, even though they involve only a small group of patients [29]. Although some may question the need for advanced research to benefit a small number of patients, it is worth remembering that the levels of prolidase activity correlate with collagen synthesis in other diseases as well, so PD research may benefit other groups of patients as well [30].

## 5. Conclusions

Due to the advanced progression of the disease, the patient required hospitalization in the ICU for a total of 83 days with highly specialized treatment and nursing care. The management of PD, as an incurable chronic disease, focused on symptom control and the management of increasing multiorgan disorders. Despite the efforts of the entire team, the effectiveness of the treatment of PD at an advanced stage was unsatisfactory. There is a need for research leading to the development of treatment guidelines for this disease.

## Figures and Tables

**Table 1:** Clinical picture and treatment used (ICU 1, 2, 3).

	Clinical Picture	Treatment/Interventions Applied
Skin symptoms	lower extremity ulcers, necrosis, hard-to-heal wounds, burns, abrasions, wound infections, high values of inflammatory parameters	targeted antibiotic therapy (Figure S1); specialized dressings (hydrocolloid, alginate, polyurethane, VAC), necrectomy, amputation of toe III of the right foot, amputation of the left lower limb above the knee—postoperative course complicated by spreading of the stump wound on the 7th day (attempted treatment with vacuum dressings), biological dressings—larval therapy in the right lower limb; HBOT (two cycles, 14 sessions), during HBOT close ENT control—numerous paracentesis treatments; decubitus prophylaxis, anticoagulation; intravenous analgesia, epidural catheter
Respiratory symptoms	ARDS, pneumonia, respiratory failure requiring mechanical ventilation	mechanical ventilation via endotracheal tube for a total of 62 days (regular tube replacement every 2 weeks), due to impaired wound healing, tracheostomy performed on day 63 (2nd day of ICU 3); broad-spectrum antibiotic therapy, antifungal treatment, mucolytic drugs (Figure S1), passive physiotherapy, VAP prophylaxis, acid–base equalization, analgosedation, steroid therapy
Hemodynamic disturbances	hypotonia, tachyarrhythmias	pressure amines by continuous infusion, antiarrhythmic drugs, β-blockers, fluid and electrolyte balance (Figure S1)
Gastrointestinal symptoms	hepatosplenomegaly	nutritional treatment (enteral and parenteral), hepatoprotective treatment, stress ulcer prophylaxis
	diarrhea (iatrogenic—antibiotic therapy, certified industrial nutrition, CDI excluded)	use of a closed set for controlled stool collection, fluid therapy according to the clinical context
	cholecystitis	cholecystectomy → during the procedure, conversion to the classical method
Urinary tract symptoms	oliguria → anuria → AKI → chronic renal failure	pharmacological stimulation of diuresis, CVVHDF, CVVHD, IHD—according to the clinical context
Autoimmune disorders	hyper IgE syndrome, hypergammaglobulinemia, AD	plasmapheresis (one cycle, five treatments every 48 h)
	microcytic anemia	Numerous transfusions of blood substitutes, and p/v treatment (Figure S1)
Infections	*Acinetobacter baumannii*—respiratory tract, wound *Enterococcus faecium VRE*—perirectal swab, urinary tract *Klebsiella pneumoniae ESBL*—wound, urinary tract, perirectal swab *Pseudomonas aeruginosa*—wound, respiratory tract, urinary tract *Morganella morganii*—wound *Candida glabrata*—urinary tract *Staphylococcus haemolyticus*—wound	broad-spectrum intravenous antibiotic therapy (Figure S1), increased epidemiological regimen, management according to the principles of asepsis and antisepsis, specialized dressings (wounds), catheter/puncture exchange, microbiological testing, closed system for controlled stool collection
	bloodstream sepsis	as above + extracorporeal blood purification techniques—CRRT with extracorporeal absorption of pro-inflammatory cytokines, local and systemic treatment (Figure S2)
	deep body temperature: 41.3 °C	external physical cooling, intravenous antipyretic treatment
Endocrinopathies	hypothyroidism	thyroid hormone treatment
Neurological symptoms	depression	patient unconscious, requiring analgesia and sedation

Abbreviations: AD—atopic dermatitis; ARDS—acute respiratory distress syndrome; VRE—vancomycin-resistant enterococcus; VAP—ventilation-associated pneumonia; HBOT—hyperbaric oxygen chamber; AKI—acute kidney injury; CVVHDF—continuous veno-venous hemodiafiltration; CVVHD—continuous veno-venous hemodialysis; IHD—intermittent hemodialysis; CRRT—continuous renal replacement therapy.

## Data Availability

No new data were created or analyzed in this study. Data sharing is not applicable to this article.

## Supplementary Materials

The following supporting information can be downloaded at: https://www.mdpi.com/article/10.3390/medicina60061006/s1, Figure S1: 1st ICU course, day 1 to 27; Figure S2: 3rd ICU course, day 64 to 83; Table S1: Laboratory test results during 1st, 2nd, and 3rd ICU hospitalization.
