# Supplementary material for: Multiorgan Failure and Sepsis in an ICU Patient with Prolidase Enzyme Deficiency—The Specificity of Treatment and Care: A Case Report
Source: Medicina (Kaunas). 2024 Jun 20;60(6):1006. doi: 10.3390/medicina60061006 (PMC11205385; doi:10.3390/medicina60061006)
Supplement: Supplementary file 1 [file medicina-60-01006-s001.zip › Table S1. Laboratory test results during 1st, 2nd, and 3rd ICU hospitalization, final.pdf]

Table S1 Laboratory test results during 1st, 2nd, and 3rd ICU hospitalization.

	1st ICU hospitalization						2nd ICU hospitalization				3rd ICU hospitalization				
	**Day**														
**Analyte**	1	8	11	15	20	22	2 (total 29)	17 (total 43)	22 (total 48)	38 (total 64)	1 (total 65)	5 (total 69)	7 (total 71)	14 (total 78)	18 (total 83)
**WBC (**x 10^3 cells/μL)	7.90	10.44	14.87	43.53	19.78	11.43	22.56	28.65	14.81	16.06	15.09	13.95	10.63	8.80	18.66
**RBC** (x 10^6 cells/μL)	2.73	3.31	3.26	4.20	2.64	3.06	2.67	3.61	2.48	2.68	2.90	2.78	2.66	2.94	2.04
**Hb** (g/dL)	7.20	8.90	8.80	11.90	7.90	9.20	7.9	10.30	7.20	7.60	8.10	7.70	7.40	8.70	5.90
**PLT** (x 10^3 platelets/μL)	79.00	197.00	425.00	486.00	186.00	111.00	93.00	346.00	123.00	111.00	95.00	85.00	56.00	26.00	26.00
**HCT** (%)	22.60	27.40	26.70	35.30	23.40	23.40	29.10	23.20	29.70	24.40	25.30	24.00	23.40	25.80	18.70
**PT** (sec)	11.00	11.60	11.90	12.00	12.60	10.70	13.10	15.30	15.80	11.40	13.00	13.00	15.00	17.00	18.60
**INR**	0.97	1.03	1.06	1.07	1.15	0.95	1.10	1.30	1.30	1.00	1.18	1.19	1.40	2.11	1.73
**APTT** (sec)	36.30	31.90	30.90	41.20	32.80	31.50	32.40	46.70	40.20	29.50	74.40	40.10	59.30	41.80	49.30
**Fg** (g/L)	4.90	–	–	–	1.10	5.60	8.63	8.30	8.02	4.76	4.60	6.60	–	4.40	–
**BUN** (mmol/L)	3.00	6.50	–	4.50	6.80	3.30	2.70	4.20	6.70	7.10	7.90	6.20	6.00	12.10	24.00
**sCr** (μmol/L)	21.00	21.00	15.00	16.00	–	13.00	41.00	49.00	64.00	64.00	73.00	66.00	79.00	69.00	159.00
**K** (mmol/L)	3.00	3.70	3.40	3.80	3.90	3.20	3.82	3.26	3.90	3.94	4.30	4.90	4.20	4.70	4.30
**P** (mmol/L)	1.14	0.94	–	1.18	–	1.14	1.84	1.68	1.10	1.38	1.08	0.84	0.96	0.93	2.40
**BG** (mmol/L)	5.40	6.50	9.10	6.60	14.10	5.20	4.90	11.70	5.80	7.90	7.40	11.30	6.20	5.20	4.10
**Serum albumin** (g/dL)	18.00	30.90	30.80	38.10	–	24.30	25.00	29.00	22.00	23.00	23.00	16.80	22.20	22.50	21.20
**ALT** (U/L)	25.00	12.00	55.00	28.00	–	92.00	25.00	83.00	205.00	307.00	203.00	120.00	62.00	18.00	14.00
**AST** (U/L)	65.00	18.00	90.00	39.00	–	112.00	23.00	182.00	158.00	515.00	238.00	158.00	85.00	67.00	21.00
**TBIL** (μmol/L)	8.00	6.70	17.00	10.00	11.00	11.30	13.50	20.40	36.30	35.10	22.91	75.20	149.00	187.50	158.60
**CRP** (mg/L)	199.74	76.28	171.49	152.05	255.65	316.31	247.80	313.60	298.00	190.20	214.08	428.76	494.27	226.93	343.50
**LAC** (mmol/L)	0.40	1.00	1.10	1.10	2.00	1.30	–	–	–	–	0.60	1.20	1.30	3.10	5.80
**PCT** (ng/ml)	0.08	0.05	0.17	0.07	2.17	0.91	0.28	1.58	5.60	0.96	1.06	10.28	33.77	16.92	8.40
**TP** (g/L)	54.60	64.00	58.20	62.10	–	50.60	67.00	78.00	92.00	83.00	75.00	68.00	68.90	56.20	49.80

Abbreviations: ALT – Alanine Aminotransferase, APTT – Activated Partial Thromboplastin Clotting Time , AST – Aspartate aminotransferase, BG – Blood Glucose, BUN – Blood Urea Nitrogen, CRP – C-Reactive Protein, Fg – Fibrinogen, Hb – Hemoglobin, HCT – Hematocrit, INR – International Normalized Ratio, K – Serum Potassium, LAC – Lactate, P – Serum Phosphorus, PCT – Procalcitonin, PLT – Platelet Count, PT – Prothrombin Time, RBC – Red Blood Cell Count, sCr – Serum Creatinine; TBIL – Total bilirubin, TP – Total Protein, WBC – White Blood Cell Count
