# Supplementary figures and images for: Multiorgan Failure and Sepsis in an ICU Patient with Prolidase Enzyme Deficiency—The Specificity of Treatment and Care: A Case Report
Source: Medicina (Kaunas). 2024 Jun 20;60(6):1006. doi: 10.3390/medicina60061006 (PMC11205385; doi:10.3390/medicina60061006)

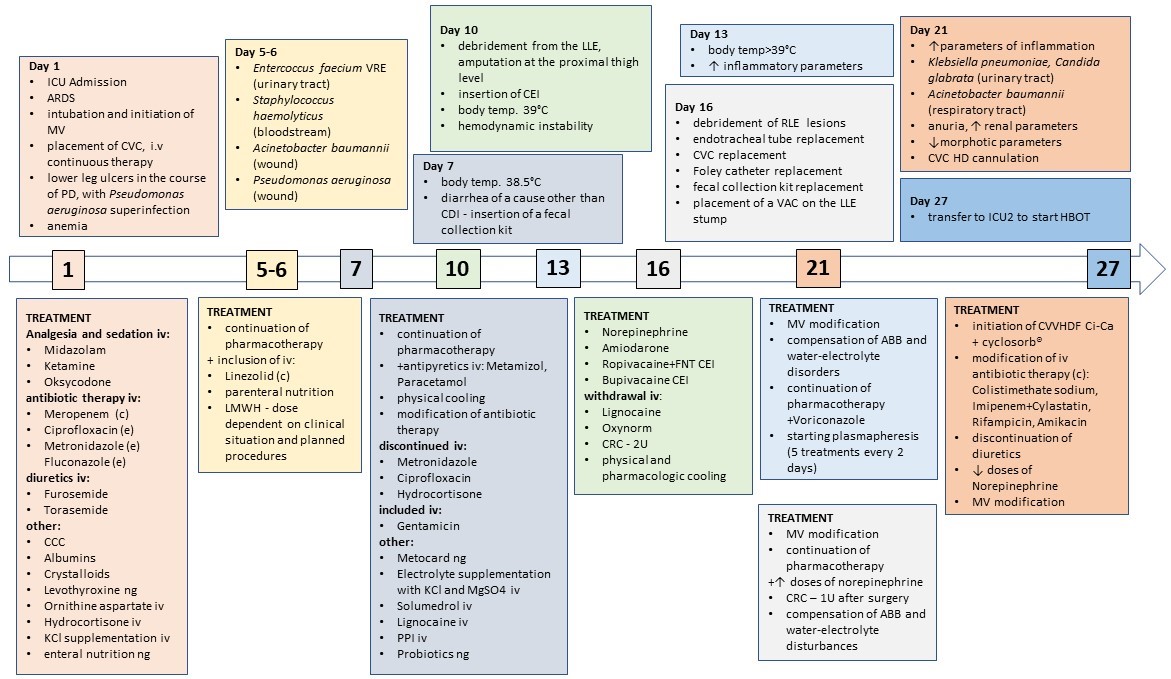

Supplement: Supplementary file 1 [file medicina-60-01006-s001.zip › Figure S1. 1st ICU course, day 1 to 27.jpg]

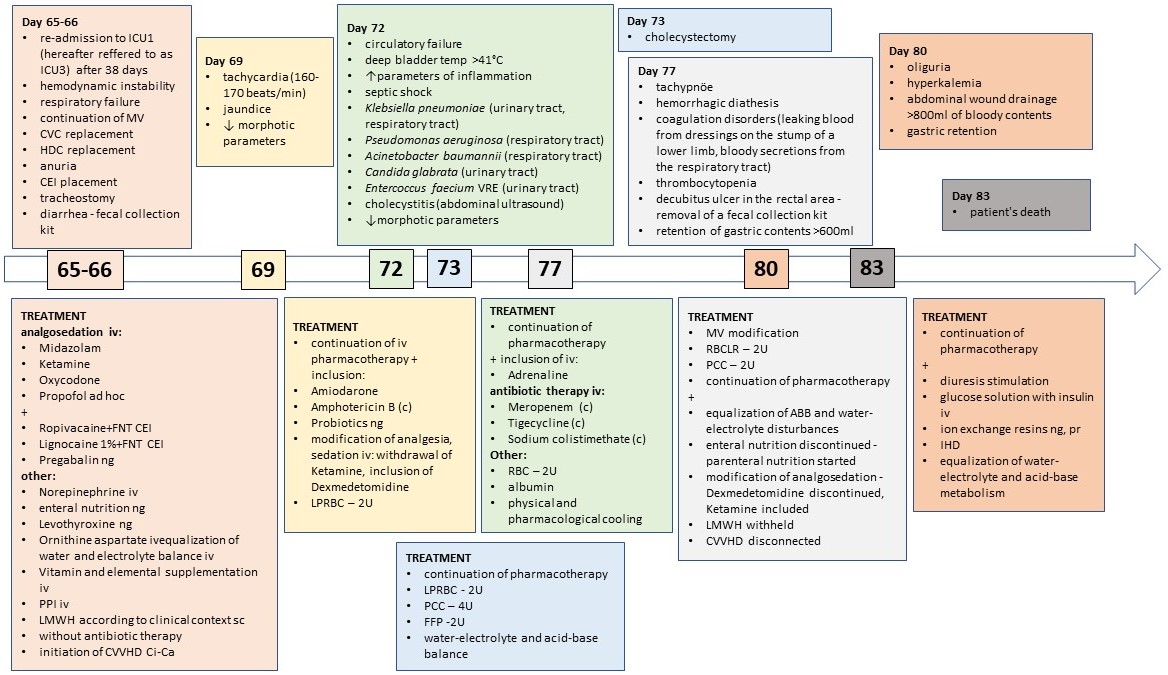

Supplement: Supplementary file 1 [file medicina-60-01006-s001.zip › Figure S2. 3rd ICU course, day 64 to 83.jpg]
